# Supplementary material for: Polymorphism within IGFBP Genes Affects the Acidity, Colour, and Shear Force of Rabbit Meat
Source: Animals (Basel). 2023 Dec 4;13(23):3743. doi: 10.3390/ani13233743 (PMC10705342; doi:10.3390/ani13233743)
Supplement: Supplementary file 1 [file animals-13-03743-s001.zip › animals-2714135-supplementary.pdf]

Table S1. *Associations between polymorphism g.3431insAC within IGFBP1and growth and slaughter traits*

Traits[3]	TW[1]					NZWxFG					PW					FG				
	GG		G/GAC		*p-value*	GG		G/GAC		*p-value*	GG		G/GAC		*p-value*	GG		G/GAC		*p-value*
	Means	SD[2]	Means	SD		Means	SD	Means	SD		Means	SD	Means	SD		Means	SD	Means	SD	
BW12	2774	54[2]	2669	53	*0.1976*	2651	43	2660	42	*0.4524*	2841	46	2834	58	*0.9734*	3194	77	3504	181	*0.1568*
SW	2808	54	2705	51	*0.2989*	2699	50	2720	49	*0.4600*	2859	46	2710	65	*0.5517*	3281	82	3693	143	*0.0713*
HCW	1494	30	1439	29	*0.1835*	1389	26	1386	27	*0.4975*	1455	25	1398	34	*0.5925*	1657	49	1898	94	*0.1329*
CCW	1478	29	1380	28	*0.1127*	1341	25	1344	26	*0.4965*	1323	21	1364	29	*0.7353*	1591	49	1832	89	*0.1559*
DPH	53.21	0.27	53.19	0.34	*0.1056*	51.30	0.28	50.90	0.29	*0.8073*	50.87	0.25	51.57	0.40	*0.9161*	50.18	0.51	51.23	0.69	*0.2818*
DPC	51.59	0.26	51.25	0.30	*0.1032*	49.48	0.26	49.34	0.27	*0.9881*	48.15	0.29	49.26	0.54	*0.7638*	48.33	0.58	49.47	0.66	*0.0820*
FMB	585	10	558	10	*0.1346*	527	11	533	11	*0.7021*	590	11	543	15	*0.4130*	688	22	791	36	*0.1822*
FF	39	6	30	3	*0.1975*	9	1	11	1	*0.4474*	26	2	15	2	*0.1146*	15	2	19	2	*0.1462*
IM	250	7	230	6	*0.2227*	239	5	237	5	*0.5925*	229	4	224	9	*0.6312*	243	9	273	21	*0.5890*
IB	**43^{a}**	**2**	**36^{b}**	**1**	***0.0025***	42	1	42	1	*0.5479*	35	1	42	1	*0.2810*	45	2	50	4	*0.4808*
IF	28	3	26	2	*0.2248*	15	1	13	1	*0.2402*	25	2	20	2	*0.4696*	13	2	19	4	*0.4629*
HM	410	7	383	7	*0.2311*	395	7	394	8	*0.5151*	397	6	372	8	*0.4903*	441	14	516	28	*0.0912*
HB	119	3	114	3	*0.8633*	106	2	109	2	*0.5437*	117	2	113	3	*0.5177*	144	4	161	6	*0.1281*
HF	4	1	4	1	*0.2362*	5	1	4	0	*0.5994*	2	0	2	1	*0.5578*	2	1	3	1	*0.2968*

[1]TW – Termond White; NZWxFG – crossbreeds of New Zealand White and Flemish Giant; PW – Popielno White; FG – Flemish Giant. [2] SD – standard deviation.[3]BW12 – body weight at 12th week of age(g);SW- Slaughter weight (g); HCW – hot carcass weight (g); FP – fore part weight (g); IP – intermediate part (loin) weight (g); HP – hind part weight (g), CCW – chilled carcass weight (g); DPH – warm dressing out percentage (%); DPC – cold dressing out percentage (%); FMB– meat and bones in fore part (g); FF - dissectible fat in fore part (g); IM - meat in intermediate part (g); IB - bones in intermediate part (g); IF - dissectible fat in intermediate part (g); HM - meat in hind part (g); HB - bones in hind part (g); HF - dissectible fat in hind part(g). [a,b] Values within a row and breeds with different superscripts differ significantly at $P<0.003$

Table S2. *Associations between polymorphism g.3431insAC within IGFBP1 and carcass traits*

Traits	TW[1]					NZWxFG					PW					FG				
	GG		G/GAC		*p-value*	GG		G/GAC		*p-value*	GG		G/GAC		*p-value*	GG		G/GAC		*p-value*
	Means	SD[2]	Means	SD		Means	SD	Means	SD		Means	SD	Means	SD		Means	SD	Means	SD	
										m. biceps femoris										
pH$_{45}$	6.57	0.06	6.75	0.06	*0.2081*	6.63	0.03	6.67	0.03	*0.5836*	6.67	0.04	6.83	0.08	*0.3895*	6.64	0.06	6.80	0.11	*0.1020*
L^*_{45}	52.49	0.49	52.46	0.47	*0.0176*	57.08	0.23	57.47	0.22	*0.0583*	50.72	0.37	52.00	1.25	*0.2858*	51.97	0.54	54.96	1.00	*0.0046*
a^*_{45}	3.07	0.13	3.83	0.23	*0.5360*	11.45	0.15	11.13	0.15	*0.0193*	2.92	0.16	3.05	0.24	*0.8723*	3.65	0.24	2.98	0.37	*0.4592*
b^*_{45}	1.01	0.29	1.10	0.29	*0.0887*	0.99	0.14	1.04	0.14	*0.2184*	0.45	0.24	0.57	0.43	*0.9651*	**1.14[a]**	**0.23**	**-0.50[b]**	**0.63**	***0.0001***
pH$_{24}$	5.85	0.03	6.11	0.06	*0.0054*	5.79	0.02	5.71	0.02	*0.0299*	5.98	0.03	5.92	0.08	*0.9774*	6.07	0.02	6.08	0.05	*0.2115*
L^*_{24}	55.36	0.44	55.20	0.37	*0.9404*	57.99	0.20	57.92	0.20	*0.0259*	56.24	0.27	54.34	0.69	*0.1652*	57.31	0.48	59.33	1.43	*0.4196*
a^*_{24}	4.18	0.24	4.84	0.32	*0.1504*	12.59	0.18	12.56	0.17	*0.2202*	3.95	0.19	4.02	0.32	*0.8211*	4.74	0.35	3.40	0.26	*0.0619*
b^*_{24}	4.21	0.32	4.29	0.27	*0.6705*	3.55	0.16	3.34	0.15	*0.5575*	4.08	0.17	3.91	0.24	*0.7067*	4.78	0.29	3.82	0.29	*0.5678*
										m. longissimus lumborum										
pH$_{45}$	6.71	0.05	6.47	0.19	*0.6576*	6.70	0.03	6.76	0.03	*0.4414*	6.66	0.06	6.48	0.10	*0.4007*	6.78	0.05	6.97	0.07	*0.1030*
L^*_{45}	59.77	0.70	58.94	0.55	*0.5776*	60.65	0.32	60.65	0.31	*0.2054*	59.43	0.64	60.24	2.23	*0.2358*	62.27	0.82	65.40	1.39	*0.0811*
a^*_{45}	0.70	0.40	1.27	0.38	*0.2488*	8.38	0.21	8.60	0.18	*0.1599*	1.55	0.41	0.13	0.86	*0.2098*	2.39	0.66	0.37	0.57	*0.0865*
b^*_{45}	-2.34	0.73	-2.30	0.50	*0.6848*	-2.59	0.15	-2.26	0.17	*0.3623*	-1.78	0.49	-2.57	0.20	*0.3635*	-1.89	0.80	-1.63	0.87	*0.5482*
pH$_{24}$	5.76	0.04	5.92	0.04	*0.1439*	5.63	0.02	5.58	0.02	*0.0306*	5.81	0.03	5.84	0.08	*0.5621*	6.03	0.05	6.06	0.08	*0.2824*
L^*_{24}	55.91	0.47	55.67	0.44	*0.1647*	56.99	0.27	56.39	0.26	*0.1164*	56.56	0.31	53.80	0.60	*0.0086*	58.76	0.62	60.92	1.77	*0.6551*
a^*_{24}	5.21	0.27	6.43	0.40	*0.0368*	12.86	0.24	13.12	0.20	*0.2042*	5.43	0.25	6.00	0.85	*0.2021*	7.88	0.40	7.01	0.61	*0.4481*
b^*_{24}	3.85	0.38	4.46	0.34	*0.2235*	1.96	0.20	1.77	0.19	*0.4057*	3.33	0.23	4.73	0.23	*0.1434*	5.76	0.42	4.95	0.75	*0.6242*
										m. longissimus lumborum										
Shear force	2.03	0.09	1.93	0.12	*0.4335*	3.79	0.12	3.98	0.13	*0.2512*	1.74	0.07	2.15	0.32	*0.0035*	2.07	0.10	2.43	0.20	*0.0926*
Hardness	13.57	0.71	13.69	0.45	*0.0542*	57.86	1.96	59.09	2.02	*0.9284*	10.76	0.39	12.87	1.04	*0.1067*	11.71	0.68	13.23	0.94	*0.6067*
Springiness	0.45	0.01	0.47	0.01	*0.5183*	0.52	0.01	0.52	0.01	*0.8015*	0.47	0.01	0.45	0.02	*0.1873*	0.49	0.01	0.49	0.01	*0.6896*
Cohesivness	0.44	0.01	0.43	0.01	*0.0357*	0.42	0.01	0.41	0.01	*0.0616*	0.43	0.00	0.43	0.01	*0.9498*	0.45	0.01	0.44	0.01	*0.3880*
Cheviness	2.74	0.19	2.81	0.14	*0.1238*	13.37	0.49	13.78	0.57	*0.7719*	2.25	0.11	2.55	0.28	*0.4311*	2.75	0.24	2.95	0.32	*0.9609*

[1]TW – Termond White; NZWxFG – crossbreeds of New Zealand White and Flemish Giant; PW – Popielno White; FG – Flemish Giant. [2] SD – standard deviation. [a,b]Values within a row and breeds with different superscripts differ significantly at *P*<0.003

Table S3. *Associations between polymorphism g.41594308T>C within IGFBP4and growth and slaughter traits*

Traits[3]	TW[1]					NZWxFG					PW					FG				
	TT		TC		*p-value*	TT		TC		*p-value*	TT		TC		*p-value*	TT		TC		*p-value*
	Means	SD[2]	Means	SD		Means	SD	Means	SD		Means	SD	Means	SD		Means	SD	Means	SD	
BW12	2778	73	2687	45	0.5254	2683	48	2647	43	0.8122	2965	49	2729	55	0.0502	3264	115	3391	127	0.5195
SW	2802	68	2740	45	0.6139	2761	55	2667	53	0.4814	2962	53	2658	56	0.0069	3337	138	3449	140	0.6118
HCW	1498	39	1456	24	0.6225	1420	30	1357	28	0.2894	**1512[a]**	**26**	**1322[b]**	**33**	**0.0028**	1657	79	1791	86	0.3085
CCW	1461	38	1410	24	0.5953	1371	29	1314	28	0.3638	1351	13	129	11	0.1749	1620	74	1694	86	0.8509
DPH	53.43	0.36	53.15	0.25	0.9139	51.41	0.35	50.85	0.30	0.2074	51.07	0.27	49.70	0.38	0.0836	49.53	0.86	51.15	0.55	0.7999
DPC	51.63	0.35	51.38	0.23	0.7826	49.61	0.33	49.20	0.29	0.4337	47.95	0.40	48.35	0.34	0.3876	48.52	1.11	48.87	0.67	0.3453
FMB	577	14	566	8	0.7287	538	12	521	12	0.5263	605	13	510	14	0.0040	692	34	737	35	0.7002
FF	39	6	30	3	0.4576	10	1	10	1	0.6131	26	3	19	3	0.1669	14	4	12	2	0.5295
IM	247	7	237	6	0.7872	242	6	233	5	0.5441	231	5	211	6	0.1735	244	14	260	16	0.9438
IB	40	2	38	1	0.5799	42	1	40	1	0.3052	35	1	30	2	0.7913	49	3	47	3	0.6691
IF	28	3	26	2	0.1514	14	1	14	1	0.7552	27	2	14	2	0.0641	13	4	14	3	0.4121
HM	405	10	394	6	0.7640	402	9	386	8	0.4039	401	7	357	8	0.0106	460	21	473	26	0.9819
HB	122	3	116	3	0.6260	107	3	106	2	0.8556	118	2	105	2	0.0072	146	5	149	6	0.6803
HF	3	1	3	1	0.1959	4	1	5	1	0.3426	3	1	1	0	0.3743	2	1	1	1	0.8750

[1]TW – Termond White; NZWxFG – crossbreeds of New Zealand White and Flemish Giant; PW – Popielno White; FG – Flemish Giant. [2] SD – standard deviation. [3]BW12 – body weight at 12th week of age(g);SW- Slaughter weight (g); HCW – hot carcass weight (gCCW – chilled carcass weight (g); DPH – warm dressing out percentage (%); DPC – cold dressing out percentage (%); FMB– meat and bones in fore part (g); FF - dissectible fat in fore part (g); IM - meat in intermediate part (g); IB - bones in intermediate part (g); IF - dissectible fat in intermediate part (g); HM - meat in hind part (g); HB - bones in hind part (g); HF - dissectible fat in hind part(g). [a,b]Values within a row and breeds with different superscripts differ significantly at $P<0.003$

Table S4. *Associations between polymorphism g.41594308T>C within IGFBP4and carcass traits*

Traits	TW[1]				*p-value*	NZWxFG				*p-value*	PW				*p-value*	FG				*p-value*
	TT		TC			TT		TC			TT		TC			TT		TC		
	Means	SD[2]	Means	SD		Means	SD	Means	SD		Means	SD	Means	SD		Means	SD	Means	SD	
										m. biceps femoris										
pH_{45}	6.59	0.06	6.63	0.06	0.4806	6.72	0.03	6.65	0.03	0.0472	6.73	0.05	6.59	0.11	0.8694	6.71	0.12	6.69	0.08	0.9006
L^*_{45}	52.80	0.43	53.03	0.50	0.8326	57.40	0.25	57.10	0.22	0.5733	50.94	0.55	49.79	0.55	0.9744	53.78	1.17	52.52	0.63	0.1206
a^*_{45}	3.01	0.25	3.51	0.22	0.8844	11.11	0.17	11.41	0.15	0.4600	2.89	0.21	3.48	0.15	0.2555	3.37	0.42	3.38	0.38	0.5392
b^*_{45}	0.87	0.35	0.96	0.23	0.5449	0.95	0.17	1.14	0.15	0.4355	0.05	0.27	1.26	0.30	0.9246	0.00	0.57	0.94	0.18	0.2187
pH_{24}	5.86	0.03	6.00	0.06	0.3424	5.80	0.02	5.76	0.02	0.0054	5.94	0.04	6.08	0.05	0.9288	6.20	0.03	6.05	0.02	0.0116
L^*_{24}	56.16	0.38	55.18	0.38	0.1693	58.31	0.20	57.58	0.23	0.1212	56.29	0.44	56.16	0.41	0.8725	56.75	0.70	57.42	0.81	0.7616
a^*_{24}	5.41	0.30	4.54	0.30	0.6195	12.25	0.20	12.81	0.18	0.1976	3.83	0.24	4.57	0.29	0.4190	4.03	0.40	4.59	0.49	0.7939
b^*_{24}	4.20	0.28	4.35	0.26	0.3641	3.43	0.18	3.65	0.14	0.3025	3.88	0.22	4.81	0.27	0.1862	4.16	0.42	4.76	0.44	0.1035
										m. longissimus lumborum										
pH_{45}	6.70	0.07	6.51	0.16	0.7121	6.79	0.03	6.74	0.03	0.0280	6.67	0.07	6.44	0.13	0.5960	6.80	0.12	6.90	0.05	0.9511
L^*_{45}	60.34	0.73	59.12	0.50	0.4806	60.84	0.35	60.31	0.33	0.5805	59.26	0.89	60.33	1.44	0.9448	62.32	1.55	63.52	1.48	0.0760
a^*_{45}	0.51	0.40	1.30	0.41	0.7207	8.44	0.22	8.70	0.20	0.8104	1.81	0.53	1.27	0.87	0.7430	3.03	1.47	1.65	0.75	0.0157
b^*_{45}	-2.54	0.66	-2.08	0.52	0.3317	-2.33	0.16	-2.22	0.18	0.0584	-1.20	0.71	-2.90	0.75	0.8840	-0.39	1.21	-0.67	0.50	0.0877
pH_{24}	5.75	0.03	5.83	0.04	0.5787	**5.66**^{a}	**0.02**	**5.60**^{b}	**0.02**	**0.0011**	5.84	0.04	5.82	0.05	0.1901	5.98	0.07	5.89	0.06	0.7830
L^*_{24}	55.11	0.40	55.44	0.41	0.4567	56.77	0.30	56.41	0.31	0.6235	56.88	0.48	55.98	0.67	0.3897	58.34	0.57	58.61	1.12	0.6886
a^*_{24}	4.34	0.22	6.17	0.41	0.8168	13.11	0.26	13.21	0.21	0.8336	5.15	0.33	6.42	0.34	0.7915	7.04	0.65	8.01	0.45	0.7032
b^*_{24}	3.96	0.35	4.05	0.34	0.3867	2.26	0.24	1.92	0.17	0.2691	3.40	0.32	3.38	0.35	0.9468	5.68	0.53	6.09	0.54	0.5245
										m. longissimus lumborum										
Shear force	2.00	0.12	1.93	0.08	0.2281	4.13	0.15	3.72	0.13	0.0203	1.79	0.11	1.88	0.15	0.5904	2.24	0.17	2.02	0.14	0.4585
Hardness	12.26	0.54	13.67	0.53	0.6510	59.75	2.07	58.75	2.10	0.4675	10.87	0.50	11.05	0.80	0.5150	12.88	0.71	11.98	1.09	0.1547
Springiness	0.45	0.01	0.46	0.01	0.4438	0.52	0.01	0.52	0.01	0.5127	0.48	0.01	0.46	0.01	0.4360	0.49	0.01	0.49	0.02	0.1300
Cohesivness	0.42	0.00	0.43	0.00	0.9725	0.42	0.01	0.43	0.01	0.0063	0.43	0.01	0.43	0.01	0.4146	0.47	0.01	0.44	0.01	0.0428
Cheviness	2.36	0.13	2.84	0.16	0.6360	13.97	0.60	13.62	0.59	0.5404	2.29	0.13	2.23	0.23	0.4408	3.02	0.27	2.70	0.37	0.0988

[1]TW – Termond White; NZWxFG – crossbreeds of New Zealand White and Flemish Giant; PW – Popielno White; FG – Flemish Giant. [2] SD – standard deviation. [a,b]Values within a row and breeds with different superscripts differ significantly at $P<0.003$

Table S5. Associations between polymorphism g.41592248A>C within *IGFBP4* and growth and slaughter traits

Traits[3]	TW[1]					NZWxFG					PW					FG	
	AA		AC		*p-value*	AA		AC		*p-value*	AA		AC		*p-value*	AA	
	Means	SD[2]	Means	SD		Means	SD	Means	SD		Means	SD	Means	SD		Means	SD
BW12	2607	41	2750	48	*0.3930*	2689	47	2648	35	*0.8570*	2889	41	2720	106	*0.0995*	3248	70
SW	2640	43	2796	45	*0.4868*	2727	56	2692	41	*0.7450*	2910	42	2703	103	*0.0648*	3348	72
HCW	1395	24	1489	25	*0.5630*	1393	31	1377	22	*0.6755*	1479	23	1400	60	*0.1429*	1699	44
CCW	1346	24	1454	25	*0.4575*	1346	30	1334	21	*0.6972*	1354	18	1269	49	*0.7504*	1637	42
DPH	52.85	0.33	53.25	0.26	*0.648*	51.01	0.32	51.01	0.24	*0.8721*	50.78	0.24	51.73	0.41	*0.4044*	50.44	0.40
DPC	50.97	0.31	51.47	0.25	*0.7707*	49.30	0.33	49.38	0.22	*0.8618*	47.99	0.28	49.75	0.27	*0.3468*	48.77	0.46
FMB	545	9	577	9	*0.5619*	529	13	526	9	*0.8689*	601	10	546	25	*0.0414*	709	18
FF	23	3	38	4	*0.3019*	10	1	10	1	*08911*	26	2	21	2	*0.5501*	15	2
IM	224	5	245	6	*0.3622*	236	5	238	4	*0.2821*	229	4	229	12	*0.5092*	248	8
IB	38	2	38	1	*0.8020*	40	1	42	1	*0.4034*	34	1	40	6	*0.6427*	46	2
IF	24	3	27	2	*0.9586*	12	1	15	1	*0.0978*	24	2	26	4	*0.7474*	14	2
HM	374	5	406	7	*0.2656*	395	9	390	6	*0.4171*	402	6	375	17	*0.0755*	456	13
HB	115	3	118	3	*0.2629*	108	2	106	2	*0.9535*	118	2	110	4	*0.0341*	147	3
HF	3	1	4	1	*0.4495*	4	1	6	1	*0.2816*	3	0	1	1	*0.2719*	2	0

[1]TW – Termond White; NZWxFG – crossbreeds of New Zealand White and Flemish Giant; PW – Popielno White; FG – Flemish Giant. [2] SD – standard deviation. [3]BW12 – body weight at 12th week of age(g);SW- Slaughter weight (g); HCW – hot carcass weight (g); CCW – chilled carcass weight (g); DPH – warm dressing out percentage (%); DPC – cold dressing out percentage (%); FMB– meat and bones in fore part (g); FF - dissectible fat in fore part (g); IM - meat in intermediate part (g); IB - bones in intermediate part (g); IF - dissectible fat in intermediate part (g); HM - meat in hind part (g); HB - bones in hind part (g); HF - dissectible fat in hind part(g). [a,b]Values within a row and breeds with different superscripts differ significantly at $P<0.003$

Table S6. Associations between polymorphism g.41592248A>C within *IGFBP4* and carcass traits

Traits	TW[1]				*p-value*	NZWxFG				*p-value*	PW				*p-value*	FG	
	AA		AC			AA		AC			AA		AC			AA	
	Means	SD[2]	Means	SD		Means	SD	Means	SD		Means	SD	Means	SD		Means	SD
m. biceps femoris																	
pH_{45}	6.66	0.07	6.63	0.05	*0.8286*	6.74	0.03	6.62	0.02	*0.1480*	6.71	0.04	6.60	0.12	*0.6560*	6.66	0.05
L^*_{45}	53.90	0.60	52.21	0.35	*0.0296*	57.23	0.26	57.34	0.18	*0.530*	50.84	0.35	50.72	1.19	*0.2885*	52.51	0.48
a^*_{45}	**4.28[a]**	**0.26**	**2.93[b]**	**0.13**	***0.0029***	11.24	0.19	11.25	0.12	*0.8620*	3.05	0.15	2.25	0.27	*0.6495*	3.55	0.22
b^*_{45}	0.69	0.29	1.05	0.24	*0.5969*	0.83	0.19	1.09	0.11	*0.5076*	0.28	0.22	1.13	0.55	*0.2230*	0.79	0.24
pH_{24}	6.08	0.07	5.88	0.03	*0.0243*	5.79	0.03	5.74	0.02	*0.5803*	5.96	0.03	6.00	0.10	*0.1267*	6.08	0.02
L^*_{24}	55.02	0.40	55.52	0.31	*0.8197*	57.79	0.26	57.99	0.15	*0.4470*	56.39	0.27	55.00	0.58	*0.1577*	57.51	0.48
a^*_{24}	4.94	0.34	4.15	0.17	*0.1618*	12.68	0.22	12.54	0.14	*0.7100*	4.01	0.17	3.15	0.32	*0.4550*	4.52	0.28
b^*_{24}	4.46	0.27	4.06	0.25	*0.7994*	3.74	0.15	3.33	0.13	*0.2431*	4.16	0.16	3.37	0.24	*0.4046*	4.57	0.23
m. longissimus lumborum																	
pH_{45}	6.39	0.20	6.73	0.03	*0.4266*	6.72	0.03	6.74	0.02	*0.0508*	6.68	0.05	6.44	0.15	*0.1704*	6.81	0.04
L^*_{45}	58.82	0.62	59.60	0.53	*0.3940*	61.11	0.39	60.15	0.24	*0.0508*	59.64	0.58	59.02	2.29	*0.0657*	62.66	0.74
a^*_{45}	2.22	0.46	0.48	0.27	*0.0852*	8.09	0.23	8.79	0.16	*0.0214*	1.77	0.40	-0.11	0.67	*0.5748*	2.23	0.56
b^*_{45}	-1.57	0.64	-2.58	0.45	*0.6808*	-2.67	0.20	-2.26	0.13	*0.0769*	-1.13	0.45	-4.75	0.42	*0.0123*	-1.55	0.64
pH_{24}	5.91	0.04	5.77	0.03	*0.2690*	5.64	0.02	5.59	0.01	*0.5330*	5.82	0.03	5.79	0.11	*0.6866*	6.01	0.04
L^*_{24}	54.51	0.48	56.38	0.34	*0.2106*	**57.26[a]**	**0.31**	**56.16[b]**	**0.21**	***0.0031***	56.55	0.30	55.06	0.88	*0.0237*	59.12	0.58
a^*_{24}	7.43	0.44	5.25	0.21	*0.0013*	12.99	0.26	13.18	0.18	*0.4487*	5.49	0.25	5.22	0.70	*0.5027*	7.65	0.34
b^*_{24}	4.89	0.31	3.99	0.29	*0.0755*	2.30	0.24	1.77	0.15	*0.2459*	3.47	0.21	3.39	0.70	*0.6161*	5.66	0.36
m. longissimus lumborum																	
Shear force	1.90	0.11	2.01	0.09	*0.0167*	**4.26[a]**	**0.15**	**3.65[b]**	**0.10**	***0.0026***	1.73	0.07	1.89	0.16	*0.4846*	2.13	0.08
Hardness	13.76	0.47	12.92	0.49	*0.1535*	60.46	2.44	57.19	1.54	*0.5238*	10.50	0.36	12.11	1.08	*0.9364*	12.18	0.53
Springiness	0.47	0.01	0.45	0.01	*0.3316*	0.53	0.01	0.52	0.01	*0.2735*	0.47	0.01	0.49	0.02	*0.8821*	0.49	0.01
Cohesivness	0.43	0.01	0.42	0.00	*0.4166*	0.43	0.01	0.41	0.01	*0.0213*	0.43	0.00	0.44	0.01	*0.4219*	0.45	0.01
Cheviness	2.87	0.15	2.54	0.13	*0.6688*	14.99	0.66	12.80	0.41	*0.0255*	2.17	0.10	2.59	0.32	*0.9700*	2.76	0.18

[1]TW – Termond White; NZWxFG – crossbreeds of New Zealand White and Flemish Giant; PW – Popielno White; FG – Flemish Giant. [2] SD – standard deviation. [a,b]Values within a row and breeds with different superscripts differ significantly at $P<0.003$

Table S7. *Associations between polymorphism g.158093018A>T within IGFBP5 and growth and slaughter traits*

Traits[3]	TW[1]				*p-value*	NZWxFG				*p-value*	PW		FG	
	AA		AT			AA		AT			AA		AA	
	Means	SD[2]	Means	SD		Means	SD	Means	SD		Means	SD	Means	SD
BW12	2704	50	2638	52	*0.3359*	2566	93	2674	33	*0.2470*	2831	37	3248	70
SW	2732	47	2705	52	*0.7550*	2582	101	2728	39	*0.1650*	2838	38	3348	72
HCW	1462	26	1420	29	*0.3692*	1319	59	1395	21	*0.1806*	1447	21	1699	44
CCW	1427	25	1369	29	*0.2442*	1271	55	1351	20	*0.1506*	1333	17	1637	42
DPH	53.51	0.23	52.49	0.37	*0.0886*	50.91	0.70	51.01	0.22	*0.8850*	50.96	0.21	50.441	0.40
DPC	51.83	0.22	50.55	0.34	*0.0119*	49.14	0.74	49.35	0.20	*0.7268*	48.43	0.25	48.768	0.46
FMB	567	10	556	11	*0.5939*	502	24	531	8	*0.2432*	584	9	709	18
FF	31	3	28	4	*0.9778*	9	2	11	1	*0.5929*	25	2	15	2
IM	245	6	225	6	*0.0447*	227	10	238	4	*0.3339*	227	4	248	8
IB	41	2	36	1	*0.0593*	41	2	42	1	*0.7829*	36	1	46	2
IF	25	2	27	3	*0.4091*	15	2	14	1	*0.4691*	25	1	14	2
HM	398	6	379	7	*0.1088*	365	16	397	6	*0.0790*	393	5	456	13
HB	117	3	114	3	*0.6145*	101	4	108	2	*0.2165*	116	1	147	3
HF	4	1	3	1	*0.1494*	6	1	5	1	*0.5725*	2	0	2	0

[1]TW – Termond White; NZWxFG – crossbreeds of New Zealand White and Flemish Giant; PW – Popielno White; FG – Flemish Giant. [2] SD – standard deviation. [3]BW12 – body weight at 12th week of age(g);SW- Slaughter weight (g); HCW – hot carcass weight (g); CCW – chilled carcass weight (g); DPH – warm dressing out percentage (%); DPC – cold dressing out percentage (%); FMB– meat and bones in fore part (g); FF - dissectible fat in fore part (g); IM - meat in intermediate part (g); IB - bones in intermediate part (g); IF - dissectible fat in intermediate part (g); HM - meat in hind part (g); HB - bones in hind part (g); HF - dissectible fat in hind part(g). [a,b]Values within a row and breeds with different superscripts differ significantly at $P<0.003$

Table S8. Associations between polymorphism g.158093018A>T within *IGFBP5* and carcass traits

Traits	TW[1]					NZWxFG					PW		FG	
	AA		AT		*p-value*	AA		AT		*p-value*	AA		AA	
	Means	SD[2]	Means	SD		Means	SD	Means	SD		Means	SD	Means	SD
						m. biceps femoris								
pH$_{45}$	6.61	0.05	6.64	0.07	*0.9608*	6.58	0.05	6.67	0.02	*0.1379*	6.69	0.04	6.66	0.05
L*$_{45}$	52.88	0.45	53.21	0.53	*0.7194*	56.75	0.44	57.48	0.17	*0.1328*	50.83	0.34	52.51	0.48
a*$_{45}$	**2.97^{a}**	**0.17**	**4.14^{b}**	**0.24**	***0.0010***	11.74	0.29	11.09	0.11	*0.0654*	2.94	0.14	3.55	0.22
b*$_{45}$	0.56	0.29	1.36	0.24	*0.0200*	1.03	0.33	1.00	0.10	*0.9894*	0.40	0.20	0.79	0.24
pH$_{24}$	**5.80^{a}**	**0.02**	**6.11^{b}**	**0.06**	***<.0001***	**5.64^{a}**	**0.03**	**5.77^{b}**	**0.02**	***0.003***	5.97	0.03	6.08	0.02
L*$_{24}$	55.13	0.38	55.27	0.30	*0.4594*	57.24	0.36	58.07	0.16	*0.0546*	56.09	0.25	57.51	0.48
a*$_{24}$	4.26	0.23	4.74	0.30	*0.2370*	13.36	0.34	12.36	0.13	*0.0094*	3.93	0.17	4.52	0.28
b*$_{24}$	4.39	0.24	4.28	0.28	*0.3692*	3.63	0.34	3.39	0.11	*0.4835*	4.05	0.15	4.57	0.23
						m. longissimus lumborum								
pH$_{45}$	6.65	0.03	6.64	0.06	*0.4999*	6.78	0.04	6.73	0.02	*0.4507*	6.63	0.05	6.81	0.04
L*$_{45}$	59.47	0.56	58.64	0.56	*0.4682*	60.41	0.60	60.71	0.25	*0.6749*	59.63	0.60	62.66	0.74
a*$_{45}$	0.89	0.38	1.47	0.38	*0.5636*	8.47	0.41	8.45	0.15	*0.9754*	1.46	0.36	2.23	0.56
b*$_{45}$	-2.06	0.62	-2.64	0.48	*0.2432*	-2.51	0.45	-2.43	0.11	*0.8716*	-1.76	0.41	-1.55	0.64
pH$_{24}$	**5.72^{a}**	**0.03**	**5.91^{b}**	**0.03**	***0.0025***	**5.52^{a}**	**0.04**	**5.62^{b}**	**0.01**	***0.0011***	5.81	0.03	6.01	0.04
L*$_{24}$	55.93	0.37	55.02	0.47	*0.3436*	55.36	0.47	56.83	0.21	*0.0123*	56.27	0.29	59.12	0.58
a*$_{24}$	5.24	0.25	6.80	0.45	*0.0135*	13.66	0.43	12.87	0.17	*0.1063*	5.49	0.23	7.65	0.34
b*$_{24}$	4.01	0.32	4.41	0.33	*0.6883*	1.44	0.31	1.88	0.15	*0.2409*	3.45	0.20	5.66	0.36
						m. longissimus lumborum								
Shear force	**2.25^{a}**	**0.10**	**1.74^{b}**	**0.08**	***0.0005***	3.30	0.21	3.95	0.10	*0.0192*	1.77	0.07	2.13	0.08
Hardness	14.13	0.63	12.70	0.35	*0.0252*	54.08	3.95	59.68	1.47	*0.1554*	10.78	0.36	12.18	0.53
Springiness	0.44	0.01	0.47	0.01	*0.0208*	0.54	0.02	0.51	0.01	*0.2095*	0.47	0.00	0.49	0.01
Cohesivness	0.43	0.00	0.43	0.01	*0.8135*	0.42	0.01	0.41	0.01	*0.5851*	0.43	0.00	0.45	0.01
Cheviness	2.80	0.17	2.62	0.12	*0.3610*	13.43	1.27	13.71	0.39	*0.7814*	2.24	0.10	2.76	0.18

[1]TW – Termond White; NZWxFG – crossbreeds of New Zealand White and Flemish Giant; PW – Popielno White; FG – Flemish Giant. [2]SD – standard deviation. [a,b]Values within a row and breeds with different superscripts differ significantly at $P<0.003$
